# Betaine Induces Apoptosis and Inhibits Invasion in OSCC Cell Lines

**DOI:** 10.3390/ijms251910295

**Published:** 2024-09-25

**Authors:** Promphakkon Kulthanaamondhita, Chatvadee Kornsuthisopon, Ajjima Chansaenroj, Ekarat Phattarataratip, Kraisorn Sappayatosok, Lakshman Samaranayake, Thanaphum Osathanon

**Affiliations:** 1 Center of Excellence for Dental Stem Cell Biology and Department of Anatomy, Faculty of Dentistry, Chulalongkorn University, Bangkok 10330, Thailand; promphakkon.k@rsu.ac.th (P.K.); chatvadee.k@chula.ac.th (C.K.); ajjima.c@chula.ac.th (A.C.); 2 Department of Oral Pathology, Faculty of Dentistry, Chulalongkorn University, Bangkok 10330, Thailand; ekarat.p@chula.ac.th; 3 College of Dental Medicine, Rangsit University, Pathum Thani 12000, Thailand; kraisorn.s@rsu.ac.th; 4 Department of Animal Husbandry, Faculty of Veterinary Science, Chulalongkorn University, Bangkok 10330, Thailand; 5 Faculty of Dentistry, University of Hong Kong, Hospital Road, Hong Kong, China; 6 Office of Research Affairs, Faculty of Dentistry, Chulalongkorn University, Bangkok 10330, Thailand

**Keywords:** oral squamous cell carcinoma, oral cancer, betaine

## Abstract

Betaine, known as trimethylglycine, is a non-toxic natural substance reported to affect cancer cell responses. This study delves into the impact of betaine on the survival, proliferation, and invasion of oral squamous cell carcinoma (OSCC) cells in vitro. Human OSCC cells (HSC-4 and HSC-7) were subjected to varying concentrations of betaine, and their viability and proliferation were assessed through colourimetric MTT and colony-forming unit assays. Cell cycle progression and cell apoptosis were also investigated using flow cytometry, while cell migration and invasion were examined using a transwell migration assay, and the mRNA expression was evaluated by a quantitative polymerase chain reaction. Finally, proteomic analysis was conducted through liquid chromatography-tandem mass spectrometry on the extracted protein component of the cells. Results indicate that betaine effectively suppressed OSCC proliferation and colony formation. It triggered early apoptosis without disrupting cell cycle progression, reduced cell migration, and inhibited invasion. Betaine exposure led to significantly decreased mRNA levels of *MMP1*, *MMP2*, and *MMP9* while downregulating *FN1*, a gene linked to epithelial-to-mesenchymal transition. Proteomic analysis revealed 9240 differentially expressed up/downregulated proteins in cells treated with betaine. The significantly upregulated proteins were associated with ATP-binding cassette (ABC) transporters, while the down-regulated proteins were associated with G protein-coupled receptors (GPCR) ligand binding. In conclusion, betaine exhibits potent anti-cancer properties by attenuating OSCC cell proliferation and mitigating invasion. Exploring this natural product as an adjunct for managing oral squamous cell carcinoma shows promise, although further investigations are needed to fully elucidate its functionality.

## 1. Introduction

Oral squamous cell carcinoma (OSCC) is linked to several risk factors, including smoking, alcohol consumption, and betel-quid chewing [1]. The primary treatment modes for the condition involve surgical resection and/or radiotherapy, leading to various side effects, such as weight loss, dysphagia, xerostomia, and depression [2]. Systemic agents used for adjuvant therapy include chemotherapeutic drugs, targeted agents, and immune checkpoint inhibitors. Furthermore, several studies have evaluated the efficacy of other natural substances, such as curcumin. However, further research is required to assess these substances. Despite the advancements in targeted cancer therapy, the prognosis of OSCC patients remains poor. For instance, a previous study has shown that the survival rate of these patients decreases with advancing age and surgical treatment, the primary therapeutic modality, yielding an overall 5-year survival rate as low as 65.1% [3]. Given the generally bleak prognosis of OSCC, early detection and targeted treatment options are crucial. In recent years, there has been a significant focus on exploring the effects of natural substances in the prevention and treatment of human diseases [4,5].

**Figure 1:**
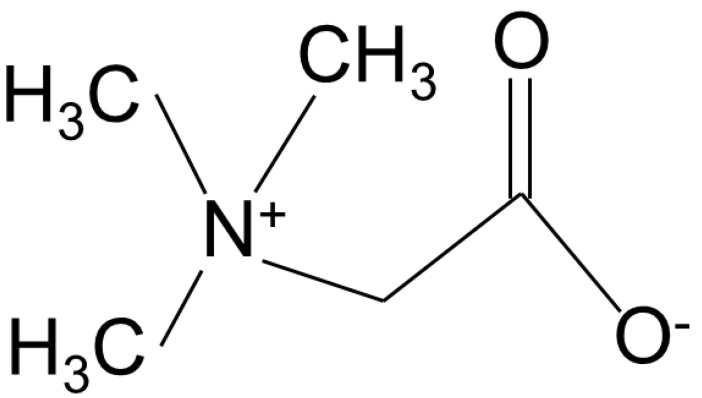
Structure of betaine. Adapted from [6].

Betaine, known as trimethylglycine, is a non-toxic natural substance that has a structural resemblance to glycine with three extra methyl groups (Figure 1). In mammals, betaine functions as an osmolyte in the kidney tissues, primarily serving to protect mammalian kidney medulla cells from osmotic stress. Its role extends to regulating concentration gradients and preventing the buildup of metabolic waste in urine. 

Another vital function of betaine is its role as a methyl group donor to the harmful metabolite, homocysteine, converting it to methionine, which is catalysed by betaine-homocysteine methyltransferase (BHMT) [6]. High levels of homocysteine, an amino acid, have been associated with an increased risk of heart disease, strokes, and other health complications. By transforming homocysteine into beneficial components, betaine contributes to maintaining healthy homocysteine levels in the bloodstream [7].

Betaine is naturally present in a variety of sources, including microorganisms, plants, and animals. Dietary sources that are rich in betaine include seafood, particularly marine invertebrates, bran or wheat germ, and vegetables, such as spinach [8]. Given its potential benefits in disease prevention, betaine is currently being investigated for its preventive and therapeutic benefits in various conditions, including cancer.

In vitro studies have demonstrated the inhibitory effects of betaine on cancer cell proliferation across several cancer types, including cervical, prostate, and breast cancers [9,10,11]. In a study involving human oral squamous cell carcinoma Cal 27 cells, betaine treatment resulted in the inhibition of cell proliferation and triggered cellular apoptosis by influencing the nuclear expression of SIRT1, a protein that plays a role in apoptotic cell death [12]. Moreover, in vivo studies in rats have indicated that betaine has the potential to impede the growth of lung cancer [13]. Moreover, inverse associations between betaine intake and cancer risks in breast, colorectal, and lung cancers have also been observed [14,15].

While previous research has delved into betaine’s impact on various cancer types, such as squamous cell carcinoma, its specific effects on oral squamous cell carcinoma, the predominant form of oral cavity cancer, remain relatively unexplored. Hence, the main aim of this study is to investigate the influence of betaine on the survival, proliferation, and invasion of human oral squamous carcinoma cells (HSC) and shed light on the putative mechanisms of its activity via proteomic analysis.

## 2. Results

### 2.1. Betaine Inhibits Proliferation and Induces Apoptosis in HSC Cells

**Figure 2:**
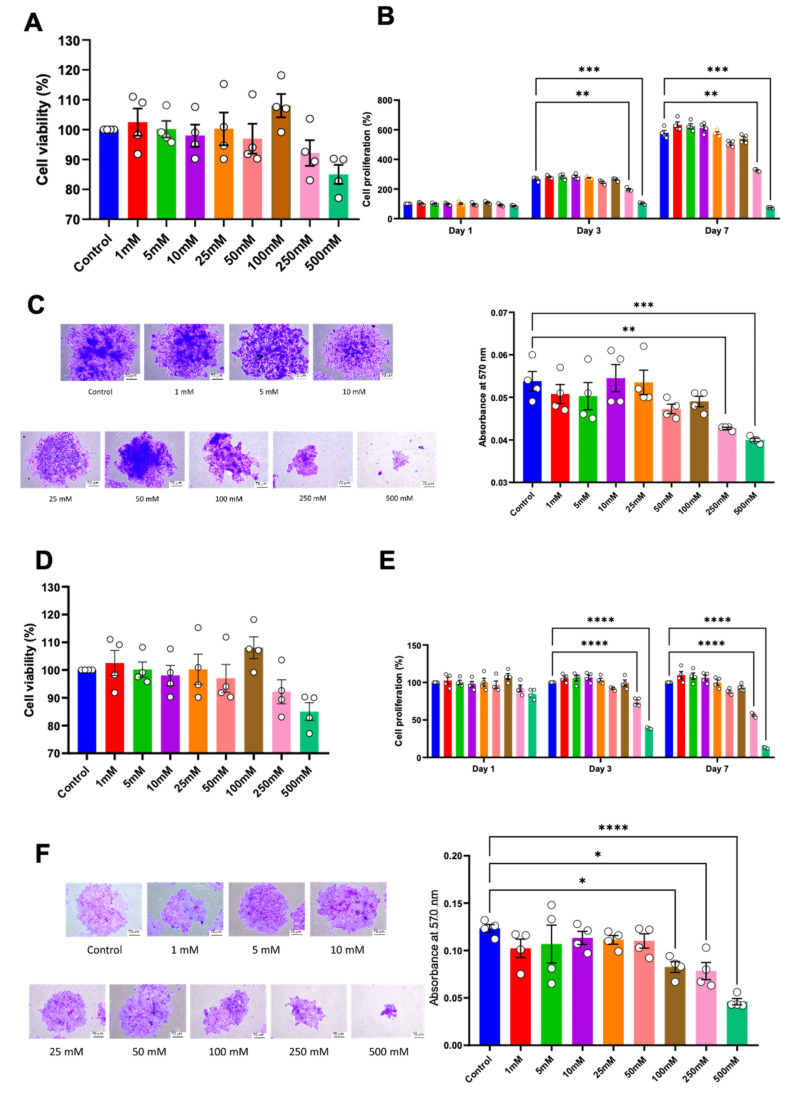
Cell viability of HSC-4 (**A**) and HSC-7 (**D**) after treatment with various concentrations of betaine for 24 h. Proliferation of HSC-4 (**B**) and HSC-7 (**E**) at 1, 3, and 7 days after betaine treatment. Colony forming unit (CFU) analysis after 14 days of betaine treatment in HSC-4 (**C**) and HSC-7 (**F**). Bar and asterisks indicate a significant difference. Each small circle represents an individual sample. The data are shown in mean ± SE, *n* = 4.

Cell proliferation was examined using a cell viability assay, colony-forming unit ability, and cell cycle analysis. No significant alteration in cell viability was observed in either HSC-4 or HSC-7 after 24 h of treatment (Figure 2A,D). However, compared to the controls, a significant reduction in cell proliferation of both cell types was observed in the 250 mM and 500 mM concentrations of betaine on day 3 and day 7 (Figure 2B,E). Correspondingly, in the colony-forming unit assay, the cells were treated with various concentrations of betaine for 14 days. The results demonstrated that betaine at a concentration of 250 mM and 500 mM significantly affected both HSC colony-forming unit size and number (Figure 2C,F).

**Figure 3:**
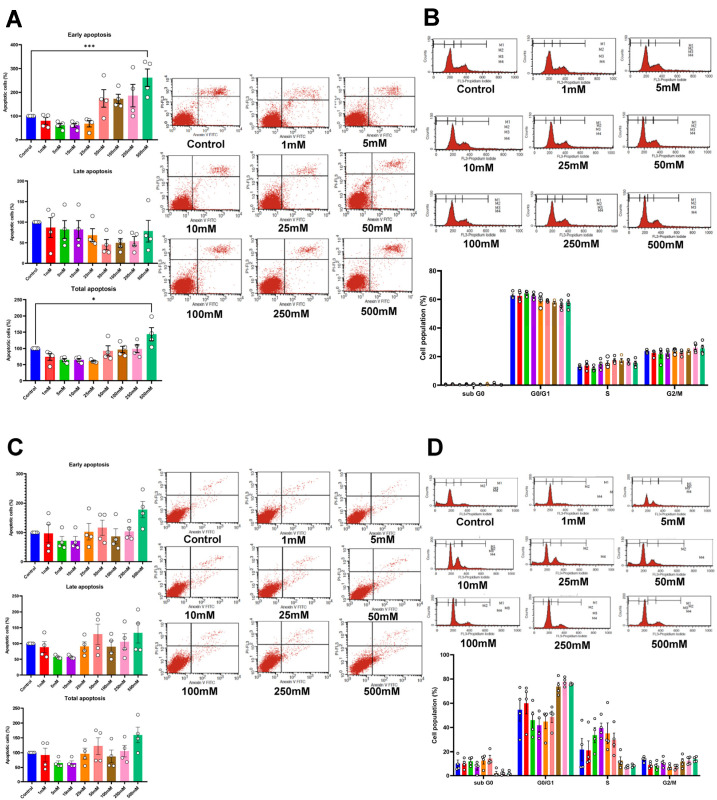
Apoptotic cell analysis using flow cytometry of HSC-4 (**A**) and HSC-7 (**C**) after 72 h of betaine treatments. Cell cycle analysis of HSC-4 (**B**) and HSC-7 (**D**) after betaine treatment for 72 h. A normal culture medium was used as a control. Bar and asterisks indicate a significant difference. Each small circle represents an individual sample. The data are shown in mean ± SE, *n* = 4.

To further evaluate the role of betaine on cell apoptosis, the cells were treated with betaine for 72 h and then stained with Annexin V FITC and PI. This led to a significant increase in early and total apoptotic cells in HSC-4, treated with 500 mM betaine (Figure 3A). Similar trends were noted in HSC-7, although they were not statistically significant (*p* > 0.05; Figure 3C). Nonetheless, the cell cycle assay at 72 h showed no significant difference in the subpopulations of cell cycles in both cell types (Figure 3B,D).

### 2.2. Betaine Suppresses the Migration and Invasion of HSC Cells

**Figure 4:**
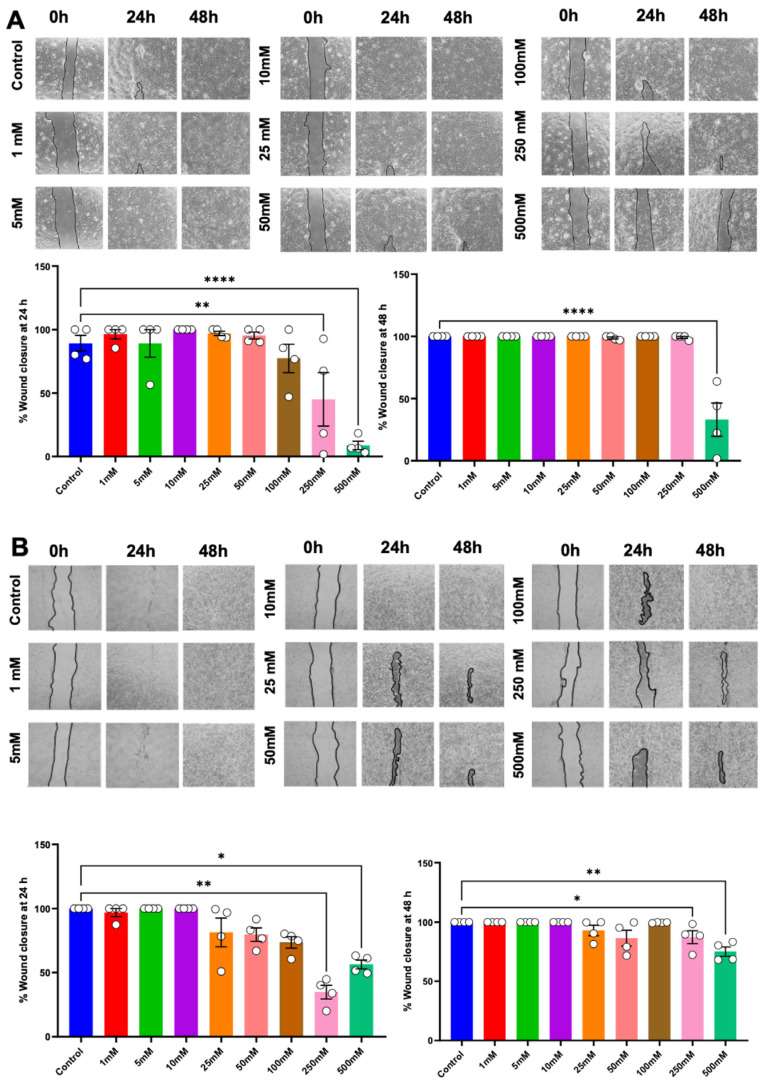
In vitro scratch assay to evaluate cell migration ability. Representative images and analysis of wound closure percentage in HSC-4 (**A**) and HSC-7 (**B**) after 24 h and 48 h of betaine treatments. A normal culture medium was used as a control. Bar and asterisks indicate a significant difference. Each small circle represents an individual sample. The data are shown in mean ± SE, *n* = 4.

An in vitro scratch assay and transwell migration assay were performed to evaluate cell migration potential. Cells were exposed to a growth medium supplemented with betaine for 24 and 48 h; cells cultured in a growth medium devoid of betaine were employed as the controls. The results revealed that betaine, at a concentration of 250 mM, led to a significant reduction in the percentage of wound closure at 24 h post-treatment. Moreover, 500 mM of betaine resulted in a significant reduction in the percentage of wound closure at both 24 h and 48 h (Figure 4A). Whereas in HSC-7, betaine at 250 mM and 500 mM concentrations of betaine markedly inhibited wound closure at both time points (Figure 4B).

**Figure 5:**
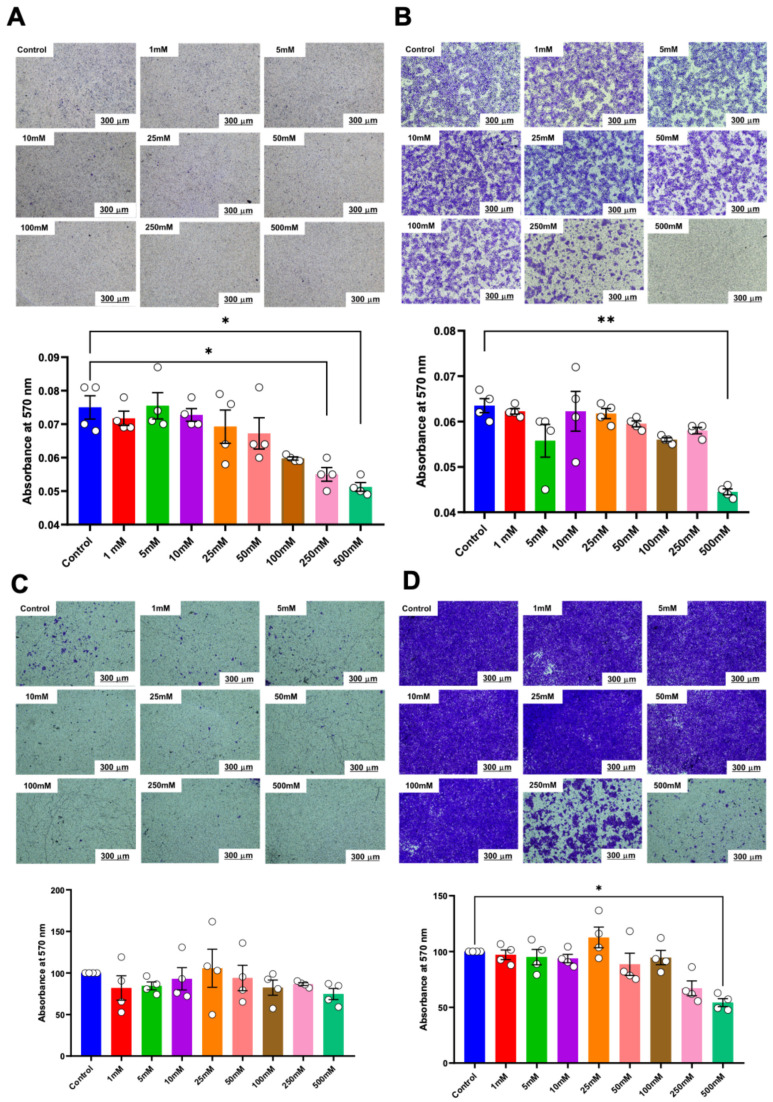
Transwell migration analysis of migrated cells. Representative images and quantitative analysis of migrated cells in HSC-4 after 24 h (**A**) and 48 h (**B**) of betaine exposure. Representative images and quantitative analysis of migrated cells in HSC-7 after 24 h (**C**) and 48 h (**D**) of betaine treatment. A normal culture medium was used as a control. Bar and asterisks indicate a significant difference. Each small circle represents an individual sample. The data are shown in mean ± SE, *n* = 4.

Similarly, in the transwell migration assay, a significant reduction in the number of migrated cells was noted in HSC-4 cells treated with 250 mM and 500 mM of betaine at both 24 h and 48 h (Figure 5A). These effects persisted up to 48 h with 500 mM of betaine (Figure 5B). In contrast, only 500 mM of betaine significantly reduced the number of migrated HSC-7 cells after 48 h of treatment (Figure 5C,D).

**Figure 6:**
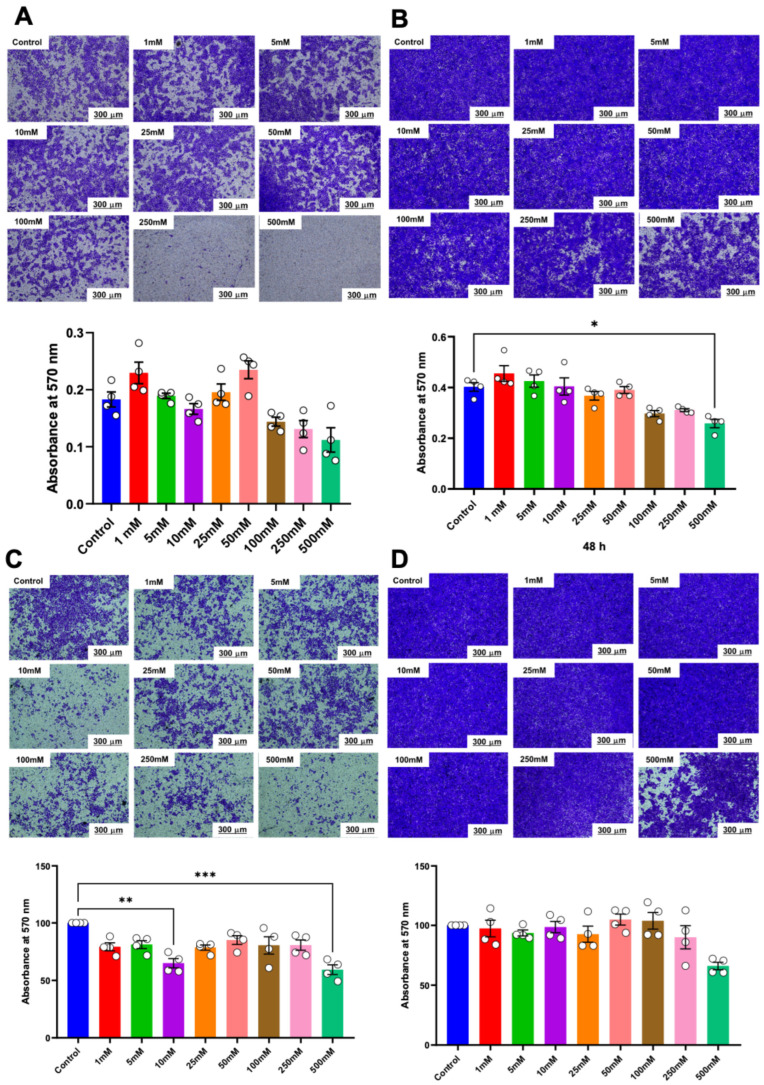
Invasion assay and quantitative analysis of the percentage of invaded cells. Representative images and quantitative analysis of invaded cells in HSC-4 after 24 h (**A**) and 48 h (**B**) of betaine exposure. Representative images and quantitative analysis of invaded cells in HSC-7 after 24 h (**C**) and 48 h (**D**) of betaine treatment. A normal culture medium was used as a control. Bar and asterisks indicate a significant difference. Each small circle represents an individual sample. The data are shown in mean ± SE, *n* = 4.

Matrigel-coated transwell plates were used to evaluate the effect of various concentrations of betaine on the invasive potential of HSC cells. The results indicated that only betaine at 500 mM was effective in reducing the invasion of HSC-4 cells at 48 h (Figure 6A,B). On the other hand, betaine at 500 mM effectively reduced the invasion of HSC-7 cells at 24 h but not at 48 h (Figure 6C,D).

### 2.3. Betaine Reduces Tumour Growth, Metastasis, and Epithelial-to-Mesenchymal Transition (EMT)-Related Genes

**Figure 7:**
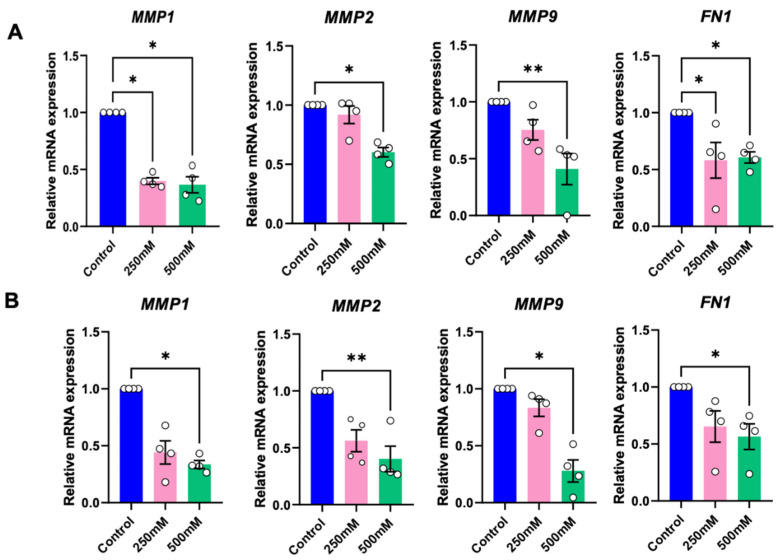
RT-PCR analysis of tumour growth, metastasis, and epithelial-to-mesenchymal transition (EMT) related genes of HSC-4 (**A**) and HSC-7 (**B**). Normal culture medium was used as a control. Bar and asterisks indicate a significant difference. Each small circle represents an individual sample. The data are shown in mean ± SE, *n* = 4.

To evaluate the influence of betaine on tumour growth and metastasis, as assessed the mRNA expression of tumour growth and metastasis-related genes, and matrix metalloproteinases (MMPs), at 24 h after treatment with betaine. Compared to the controls, betaine at 250 mM significantly reduced the mRNA expression of *MMP1* in HSC-4 cells, and conversely, 500 mM betaine significantly decreased *MMP1*, *MMP2*, and *MMP9* mRNA expression in both cell types (Figure 7A,B).

Fibronectin (*FN-1*), a key gene found to be upregulated in the epithelial-to-mesenchymal transition (EMT) process, was used to evaluate the influence of betaine on EMT. Both 250 mM and 500 mM of betaine demonstrated a significant reduction of *FN1* mRNA expression in HSC-4 (Figure 7A), while in HSC-7, only 500 mM of betaine significantly suppressed the *FN1* mRNA expression (Figure 7B).

### 2.4. Proteomics Analysis of Differentially Expressed Protein

**Figure 8:**
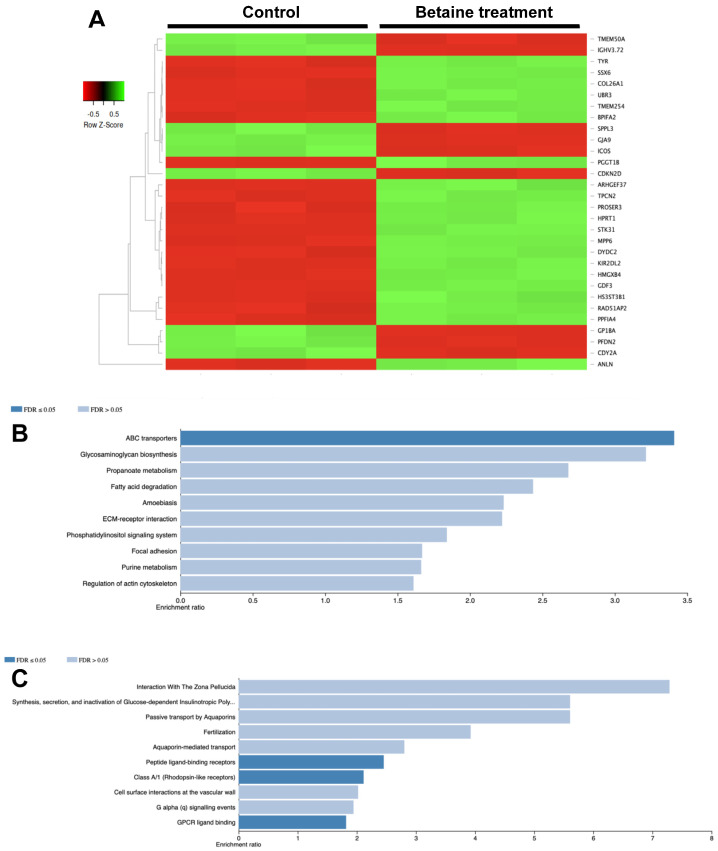
Proteomic analysis of HSC-4 cells after 500 mM betaine treatment. Heatmap of top 30 differentially expressed proteins (**A**). KEGG enrichment analysis of significantly upregulated proteins (**B**) and significantly downregulated proteins (**C**).

The differentially expressed proteins after the betaine treatment were evaluated and compared with the untreated controls. The top 30 differentially expressed proteins so derived are illustrated in Figure 8A. Enrichment analysis using the KEGG database revealed that these significantly upregulated proteins were associated with the ATP-binding cassette (ABC) transporters (Figure 8B). On the other hand, the significantly downregulated proteins were shown to participate in peptide ligand-binding receptors and G protein-coupled receptors (GPCR) ligand binding (Figure 8C).

## 3. Discussion

The use of natural compounds as an alternative treatment for diseases with high mortality rates, such as cancers, has garnered significant interest in the research community. Several in vitro and in vivo studies have shown the anti-cancer effects of betaine, such as its ability to impede cancer cell proliferation and metastasis and trigger apoptosis in cancer cells [9,13,16]. Nonetheless, its specific impact on oral cancer, especially squamous cell carcinoma, remains little explored.

We demonstrate here that 500 mM betaine effectively restrained HSC proliferation by inducing early apoptosis in HSC-4. Furthermore, it diminished HSC-4 cell migration, suppressed invasion, and downregulated the expression of mRNA associated with tumour growth, metastasis, and epithelial-to-mesenchymal transition. Additionally, proteomic analysis unveiled differentially expressed proteins linked to tumorigenesis, presenting potential targets for cancer therapies.

Cell proliferation is a crucial factor associated with malignancy, and interventions aimed at inhibiting this process play a critical role in cancer management. Previous studies utilising betaine have shown its effects in reducing cell proliferation with or without interfering with cellular apoptosis or inducing cell cycle arrest [10], results that are similar to ours. Thus, in a study by D’Onofrio et al., the combined effects of δ-valerobetaine (δVB) and γ-Butyrobetaine (γBB) were explored on various head and neck squamous cell carcinoma cell lines. Their findings revealed marked inhibition of cell proliferation, particularly in those of the Cal 27 lineage. Furthermore, temporal induction of apoptosis and G2/M arrest were also observed, suggesting that cell proliferation was attributable to both cellular apoptosis and cell cycle arrest mechanisms [12].

In a separate investigation, Bingula et al. observed similar outcomes concerning cell proliferation and cell cycle arrest in the G2/M phase following combined betaine and C-phycocyanin (C-PC) treatment of the lung cancer A459 cell line. Compared with our study, where we found that betaine suppressed cell proliferation and induced early cellular apoptosis in OSCC without affecting the cell cycle, these results underscore apoptosis as the primary mechanism responsible for repressing cell proliferation [13].

Our study noted a notable reduction of *MMP-1*, *MMP-2*, and *MMP-9* mRNA expression levels post-betaine treatment. Previous research has highlighted the pivotal role of matrix metalloproteinases (MMPs) in modulating the tumour microenvironment, particularly in orchestrating the invasion and metastasis of oral squamous cell carcinoma [17,18]. Additionally, other studies have indicated a correlation between higher OSCC histopathological grade and increased MMP-1 protein expression [17]. Moreover, the upregulation of MMP-2 and MMP-9 has been consistently reported in OSCC patients with lymph node metastasis [18,19]. Therefore, overexpression of MMP-2 and MMP-9 may serve as indicators of metastatic phenotype, suggesting that monitoring changes in MMP expression following treatment could be a valuable marker for assessing treatment outcomes in patients with oral cancer [20].

Epithelial-mesenchymal transition is a biological process that permits a polarised epithelial cell to undergo biochemical changes leading to transition into mesenchymal cell phenotypes. This transformative process entails enhancing migration, leading to increased invasive ability, apoptotic resistance, and extracellular matrix (ECM) production [21]. Fibronectin, a gene upregulated during the epithelial-to-mesenchymal transition process, is a high molecular weight glycoprotein in the ECM [22]. Its role in several biological processes, including cell proliferation and adhesion, has been well documented [23]. It is known that in head and neck squamous cell carcinoma, fibronectin is notably overexpressed and linked to advanced pathological stages and poor prognosis [22]. In vitro experiments have shown that downregulation of fibronectin could suppress the proliferation, migration, and invasion of HNSCC cells while also inhibiting the macrophage M2 polarisation [24]. Our study revealed that betaine could effectively suppress the expression of fibronectin, potentially contributing to the inhibition of cancer cell proliferation and invasion.

Multidrug resistance (MDR) characterises the ability of target cells to resist a variety of structurally and mechanically unrelated drugs, for example, by effluxing these agents across target cell membranes. This phenomenon can lead to the failure of cancer chemotherapy as cancer cells effectively reduce the intracellular levels of chemotherapeutic agents [25]. ATP binding cassette (ABC) transporters have been found to overexpress in tumours exhibiting MDR, suggesting their involvement in this resistance mechanism [26]. Multiple efforts have been taken to counteract this resistance, with the aim of enhancing the effectiveness of standard chemotherapeutic anti-cancer drugs by combining them with ABC transporter modulators [27]. We report here that the proteins associated with the ABC transporter were overexpressed following betaine exposure. However, the specific modulatory effects on the ABC transporter, whether they involve enhancing or reducing its function, require further clarification. Additional research is necessary to delve deeper into these interactions and their implications.

G protein-coupled receptors (GPCRs) are a large and diverse family of signalling receptors that play a crucial role in cell growth through the activating multiple mitogen-activated protein kinases (MAPKs) cascades. They can stimulate second messengers and trigger various downstream cascades, both in the cytoplasm and nucleus, thereby modulating normal cell functions [28]. Cancer cells often exploit these pathways to aid tumorigenesis, angiogenesis, invasion, and metastasis [29]. In the context of our study, we noted that GPCR ligand binding was associated with significantly downregulated proteins following betaine treatment. This finding suggests that targeting GPCRs could hold promise for combating drug resistance in cancer, which is a major hurdle in cancer therapeutics. However, the precise mechanisms by which betaine exerts its effects—whether it directly targets GPCRs or selectively modulates downstream signalling molecules require further investigation to elucidate its potential in developing effective cancer therapies.

Studies have shown heightened invasion and metastatic potential in metastasised cell lines. Therefore, the efficacy of a given treatment regimen may differ between the primary and metastatic tumour sites [30]. In this study, both the HSC-4, the metastasised SCC extracted from the lymph node, and HSC-7, a primary SCC from the tongue cell lines, were employed. As OSCC most predominantly affects the lateral/ventral tongue [31,32], it was deemed appropriate to examine the impact of betaine on HSC-7 cancer cells derived from this specific site. Moreover, as HSC-4 cells metastasised from the tongue, they serve as surrogate cancer cells from one of the most common sites of OSCC. Our results demonstrate comparable effects across both cell lines, suggesting that betaine may offer potential benefits in managing both primary and metastasised tumours.

Our study has a few limitations. We utilised the anchorage-colony forming ability to examine the ability of betaine to inhibit the colony formation of the cancer cells. This method primarily reflects cellular abilities for anchorage-dependent growth, a trait typical of normal cells [33]. Since cancer cells have the capability to grow and divide without binding to a substrate, considered a hallmark of carcinogenesis [34,35], the use of anchorage-independent colony formation assays could provide a more comprehensive evaluation of betaine’s effects.

Finally, we used relatively high concentrations of betaine which could potentially have cellular toxicity to the normal cells. It is crucial to evaluate the toxicity using normal oral keratinocytes before developing these substances for therapeutic uses. Recent data from our publication investigating the effects of betaine on dental pulp cells found no cellular toxicity of betaine concentrations below 250 mM [36]. This suggests the importance of further assessing the dosage and potential cytotoxic effects of betaine on various cell types to ensure its safety and efficacy in a therapeutic context.

## 4. Materials and Methods

### 4.1. Oral Squamous Cell Carcinoma Cell Lines

The study protocols were approved by the Human Research Ethics Committee of the Faculty of Dentistry, Chulalongkorn University (HREC-DCU 2022-101). HSC-4 cell lines used in the study were purchased from RIKEN BioResource Research Center, Ibaraki, Japan (RRID:CVCL_1289), and HSC-7 cell lines (RRID:CVCL_A618) were provided by Professor Teuro Amagasa, Tokyo Medical and Dental University, Tokyo, Japan [37]. The cells were cultured with Dulbecco’s Modified Eagle Medium (DMEM, Gibco, New York, NY, USA) supplemented with 10% fetal bovine serum (FBS, Gibco, New York, NY, USA), 250 ng/mL amphotericin B (Antibiotic–Antimycotic, Gibco, New York, NY, USA), 100 unit/mL penicillin, 100 µg/mL streptomycin and 2 mM L-glutamine (GlutaMAX-1, Gibco, New York, NY, USA). The culture medium was changed every 48 h. After an 80% confluent monolayer of cells was reached, the cells were trypsinised, washed, and then subcultured to new tissue culture disks and used for further experiments.

### 4.2. Preparation of Betaine Solution

Betaine (Sigma Aldrich, Burlington, MA, USA) was dissolved in sterile distilled water and aliquoted into 1 mM, 5 mM, 10 mM, 25 mM, 50 mM, 100 mM, 250 mM, and 500 mM dilutions. The latter betaine solutions were appropriately diluted with the culture medium and used in the experiments. A Betaine-free culture medium was used as the negative control.

### 4.3. Cell Viability Assay

The colourimetric MTT (3-[4,5-dimethylthiazol-2yl] diphenyltetrazolium bromide) assay was used to determine cell viability. The cells were seeded into 96-well plates at a density of 5 × 10^4^ cells/well and allowed to attach overnight, after which the medium was refreshed with varying concentrations of betaine and cultured for 24 h. The medium was then removed, and 0.5 mg/mL of MTT solution was added and incubated for 1 h at 37 °C. The resulting formazan crystals were dissolved using a formazan lysis solution. The optical density was measured at 570 nM with a microplate reader (Molecular Devices, Palo Alto, CA, USA). MTT assay was also employed to evaluate cell proliferation.

### 4.4. Colony Forming Unit (CFU) Assay

For the CFU assay OSCC cells were cultured (500 cells/well in a 6-well plate) with a medium containing various concentrations of betaine as described above. After a two-week culture period, the cells were fixed with 4% buffered formalin for 10 min and stained with crystal violet (Sigma-Aldrich, Burlington, MA, USA). An inverted microscope (Olympus, Breinigsville, PA, USA) was used to quantify CFUs. The combination of 5% (*v*/*v*) methanol and 7.5% (*v*/*v*) acetic acid solution was used to elute the staining, and the absorbance was read at 570 nM using a microplate reader.

### 4.5. Cell Cycle and Apoptosis Analysis

For cell cycle analysis, cells (1 × 10^5^ cells/well in a 12-well plate) were cultured in a medium containing various concentrations of betaine as described above. After 72 h, the cells were trypsinised and fixed in 70% ethanol at −20 °C for 30 min. Two µL of 4 mg/mL RNase A (Thermo Fisher Scientific, Waltham, MA, USA) was added to eliminate RNA. Then, the cells were stained with 40 µg/mL propidium iodide and analysed using a FACS^Calibur^ flow cytometer (BD Bioscience, Bergen, NJ, USA).

Apoptosis was evaluated using an Annexin V-FITC antibody (BD Biosciences) and propidium iodide (Sigma-Aldrich, Burlington, MA, USA). Cells were seeded into 24-well plates at a density of 1 × 10^5^ cells/well and allowed to attach overnight. Then, cells were stimulated with various betaine concentrations for 24 h before subsequent analysis. Cells were trypsinised and collected for apoptosis detection using Annexin V-FITC Apoptosis Detection Kit (eBioscience, San Diego, CA, USA).

### 4.6. Migration and Invasion Assay

The in vitro scratch assay and Transwell^®^ assay were performed to determine cell migration. For the scratch assay, the cells were plated and cultured till confluence in 60-mm culture dishes. A linear scratch was made on the cell monolayer using a 200 µL pipette tip. Scratched cells were washed with PBS to remove the debris, and the remaining cells were stimulated with various concentrations of betaine for 24 h. To quantify cell migration, phase-contrast images of the scratched area were obtained, and the cells that migrated into the scratched area at 0, 24 h, and 48 h after the scratch event were quantified.

For the Transwell^®^ migration assay, commercially available Transwell^®^ polycarbonate membrane cell culture inserts in 24-well plates (Sigma-Aldrich, Burlington, MA, USA) were used. Cells were seeded into either 24-well inserts or Matrigel-coated invasion chambers at 1 × 10^5^ cells per well. Inserts were placed into 24-well plates containing various concentrations of betaine. After 24 h and 48 h of incubation, non-migrated cells in the top chamber were removed with a cotton swab. Cells on the underside of the membranes were fixed with paraformaldehyde and stained with crystal violet. The combination of 5% (*v*/*v*) methanol and 7.5% (*v*/*v*) acetic acid solution was used to elute the staining. The absorbance was read at 570 nM using a microplate reader.

### 4.7. Quantitative Real-Time Polymerase Chain Reaction (qRT-PCR)

TRIzol reagent (Invitrogen, Carlsbad, CA, USA) was used to extract the total cellular RNA. The extracted RNA was then treated with RNase-Free DNase I (Qiagen, Venlo, The Netherlands). A nanodrop spectrophotometer (Thermo Scientific, Massachusetts, USA) was used to evaluate the amount of RNA and its integrity. A reverse transcriptase ImPromII kit (Promega, Madison, WI, USA) was used to convert 1 microgram of total RNA to complementary DNA (cDNA). A real-time polymerase chain reaction was performed using a FastStart Essential DNA Green Master kit (Roche Diagnostic, Indianapolis, IN, USA) on a MiniOpticon real-time PCR system (Bio-Rad, Berkeley, CA, USA). The real-time polymerase chain reaction began with denaturing at 95 °C for 5 min, subsequently by 40 cycles of amplification, and finished with an extension reaction at 72 °C for 20 min. The amplification cycles were 95 °C for 20 s, followed by 60 °C for 20 s, and ended with 72 °C for 20 s, respectively. The mRNA levels of target genes were normalised to succinate dehydrogenase A (*SDHA*), and relative gene expression was quantified by the comparative Ct method (2^−ΔΔCt^ method). The genes used for analysis are listed in Table 1.

### 4.8. Proteomics Analysis

After 24 h of culture with 500 mM of betaine, HSC-4 cells were trypsinised and washed using PBS solution. Cell pellets were collected, and proteomic analysis was performed using liquid chromatography-tandem mass spectrometry. Bioinformatic analysis was performed using the Reactome database with an adjusted false discovery rate of 0.05. A heatmap of significantly altered proteins was generated using the Heatmapper software version 1.0 [42]. The associated pathways of the differentially expressed proteins were assessed by WebGestalt (WEB-based Gene SeT AnaLysis Toolkit) [43] using the Kyoto Encyclopedia of Genes and Genomes (KEGG) [44] and Reactome databases [45].

### 4.9. Statistical Analysis

Data were analysed using Kruskal-Wallis tests followed by a pairwise comparison test to evaluate differences between experimental groups using GraphPad Prism version 9.1.1 (GraphPad Software, San Diego, CA, USA). *p*-values > 0.05 were considered statistically significant differences.

## 5. Conclusions

Betaine is an important natural substance that exhibits anti-cancer properties. Betaine mainly functions by suppressing cancer cell proliferation, migration, and invasion as well as associated with metastatic processes and alteration in epithelial to mesenchymal transition properties. With the limitation of the present study, betaine potentially demonstrated positive functions in the oral squamous cell carcinoma treatment. Considering the properties of betaine on cancer cells, developing this natural product as an adjuvant treatment modality is of great interest. However, the exact biological function of betaine remains to be further investigated.

## Figures and Tables

**Table 1:** The oligonucleotide sequences.

Gene of Interest	Oligonucleotide Sequence	Reference
*MMP-1*	FW: 5′-GGCCCACAAACCCCAAAAG-3′ RW: 5′-ATCTCTGTCGGCAAATTCGTAAGC-3′	[38]
*MMP-2*	FW: 5′-TTGCCATCCTTCTCAAAGTTGTAGG-3′ RW: 5′-CACTGTCCACCCCTCAGAGC-3′	[39]
*MMP-9*	FW: 5′-TGCCCGGACCAAGGATACAGTTT-3′ RW: 5‘-AGGCCGTGGCTCAGGTTCAGG-3′	[38]
*FN1*	FW: 5′-TCGAGGAGGAAATTCCAATG-3′ RW: 5′-ACACACGTGCACCTCATCAT-3′	[40]
*SDHA*	FW: 5′-CATCCACTACATGACGGAGCA-3′ RW: 5′-ATCTTGCCATCTTCAGTTCTGCTA-3′	[41]

## Data Availability

The data that support the findings of this study are available from the corresponding author upon reasonable request. The mass spectrometry-based proteomics datasets generated during and/or analysed during the current study are available in the PRIDE repository (accession no PXD054595).
